# Importance of the GP–patient relationship

**DOI:** 10.1080/13814788.2019.1679469

**Published:** 2019-10-30

**Authors:** Stéphane Henninger, Brenda Spencer, Olivier Pasche

**Affiliations:** a ForOm Nord Vaudois, Yverdon, Switzerland;; b Institute of Social and Preventive Medicine (IUMSP), Lausanne University Hospital, Lausanne, Switzerland;; c Department of Ambulatory Care and Community Medicine, University of Lausanne, Lausanne, Switzerland

We thank our colleagues Philips et al., for their comments and for sharing their interesting research programme [1]. For us, it seems important to highlight that although availability was an issue, our principal conclusion is the importance of the GP–patient relationship [2]. Our results show how this is a cornerstone for the patient in deciding whether to consult an emergency department (ED) or their GP.

Philips et al. [1] write that patients appreciate the availability of their GP but making primary caregivers more available will not be sufficient to control ED overload. This problem must be addressed in a variety of ways; nevertheless, we believe that having more GPs available, as they know their patients personally, could be one effective solution. Implementing out-of-hours GP cooperatives could decrease the number of consultations in ED. However, it is often the patients with the poorest social integration who use emergency services instead of consulting a GP. Moreover, out-of-hours GP cooperatives do not necessarily imply good doctor–patient communication and are therefore not an alternative to the establishment of an ongoing relationship between the GP and their patient as we have described.

We believe that the physician’s personal commitment to his or her patient is probably one of the most important determinants of the patient’s sense of safety, and this has a major impact on his or her decision to consult an ED. This point is illustrated by our participants who cited how grateful they were when the GP gave his or her private phone number, even if they did not use it. To limit excessive use of the ED, our results indicate the need to maintain a strong primary care system providing not only sufficient GP availability but also emphasizing the quality of the doctor–patient relationship.
